# Comparative analysis of prevalence, evaluation, management, and rehabilitation outcome of spontaneous isolated visceral artery dissection: a systematic review and meta-analysis of 80 reports

**DOI:** 10.1097/JS9.0000000000000301

**Published:** 2023-03-24

**Authors:** Yan Shi, Jiangzhou Guo, Jige Dong, Xueli Chen, Lihua Luo, Yan Shen

**Affiliations:** Departments of a Rehabilitation and Treatment; b Rehabilitation, Wangjing Hospital, China Academy of Chinese Medical Sciences, Wangjing Hospital; c Department of Rehabilitation Medicine, Beijing Shijitan Hospital Affiliated to Capital Medical University; d Rehabilitation Center; e Physical Therapy Center, Wangjing Hospital of CACMS, Beijing, China

**Keywords:** isolated visceral artery dissection, management, prevalence

## Abstract

Because of relatively little data for management and evaluation surrounding spontaneous isolated visceral artery dissection (IVAD), existing studies have failed to provide comprehensive analysis for the management, evaluation, prevalence, as well as natural course of the disease. Therefore, we collected and analyzed current evidence on spontaneous IVAD with the aim of providing quantitative pooled data for the natural course and treatment standardization of the disease.

**Methods:**

A systematic search of PubMed, Embase, the Cochrane Library, and Web of Science up to 1 June 2022, was conducted for relevant studies that investigating the natural course, treatment, classification, and outcomes of IVAD. The primary outcomes were to determine the difference in prevalence, risk factors, and characteristics between different spontaneous IVAD. Two reviewers assessed the trial quality and extracted the data independently. All statistical analyses were performed using the standard statistical procedures provided in Review Manager 5.2 and Stata 12.0.

**Results:**

A total of 80 reports with 1040 patients were identified. The pooled results indicated that in IVAD, isolated superior mesenteric artery dissection (ISMAD) was more prevalent, with a pooled prevalence of 60% (95% CI: 50–71%), followed by isolated celiac artery dissection (ICAD) (prevalence: 37%; 95% CI: 27–46%). IVAD was male predominated with a pooled proportion of 80% (95% CI: 72–89%). Similar results were found in ICAD (prevalence: 73%; 95% CI: 52–93%). More IVAD patients were diagnosed with symptoms than ICAD (64 vs. 59%). Regarding to the risk factors, this pooled analysis found smoking and hypertension were the top two conditions in both spontaneous IVAD and ICAD patients, with proportion of 43, 41, 44, and 32%, respectively. It was observed that ICAD appeared shorter dissection length (mean difference: −3.4 cm; 95% CI: −4.9 to −2.0; *P*<0.0001), higher prevalence of Sakamoto’s classification Π (odds ratio: 5.31; 95% CI: 1.77−15.95; *P=*0.003) and late progression (odds ratio: 2.84; 95% CI: 1.02−7.87; *P=*0.05) than ISAMD.

**Conclusions:**

Spontaneous IVAD was male predominant and ISMAD was most prevalent followed by ICAD. Smoking and hypertension were the top two conditions in both spontaneous IVAD and ICAD patients. The majority of patients diagnosed with IVAD received observation and conservative treatment and experienced a low proportion of reintervention or progression, especially for ICAD patients. In addition, ICAD and ISMAD had several differences in clinical features and dissection characteristics. Future studies with enough sample size and long follow-up are required to clear the management, long-term outcome, and risk factors of the IVAD prognosis.

Highlights

- Spontaneous isolated visceral artery dissection (IVAD) was male predominant and isolated superior mesenteric artery dissection was most prevalent followed by isolated celiac artery dissection (ICAD).
- Smoking and hypertension were the top two conditions in both spontaneous IVAD and ICAD patients.
- Observation and conservative treatment were applied to the majority of IVAD and experienced a low incidence of reintervention or progression, especially for ICAD patients.
- ICAD and isolated superior mesenteric artery dissection had several differences in clinical features and dissection characteristics.

## Introduction

Isolated visceral artery dissection (IVAD), always including dissections locating in the celiac artery, superior mesenteric artery, inferior mesenteric artery, and their respective branches, was a rare clinical condition. Recently, spontaneous IVAD has been detected more frequently regardless of the presence or absence of symptoms because of the increased use of computed tomography for evaluating abdominal pain and of advances in diagnostic tools^1^–^3^. The short-term complications of IVAD include organ ischemia necrosis and dissection rupture, while the long-term complications include dissection aneurysm rupture and hemorrhage, which can endanger life of patients^4^,^5^. At present, the pathogenesis of IVAD is still unclear, and the best treatment is still controversial. Many reports suggested conservative treatment as the optimal choice because the clinical symptoms of most patients can be relieved^6^–^9^. However, the dissection progression and dissection aneurysm formation that occurred after conservative treatment of some IVAD may make these patients miss the best intervention opportunity and increase the difficulty of later treatment^10^–^12^.

Spontaneous IVAD is often displayed as multiple locations of dissection. Isolated celiac artery dissection (ICAD) is always co-existed with other visceral artery dissections, such as the dissection in superior mesenteric artery, left gastric artery, splenic artery, common hepatic artery, and gastroduodenal artery^9^,^12^–^15^. Spontaneous ICAD and isolated superior mesenteric artery dissection (ISMAD) represent the dominant types of IVAD^16^. Although the incidence was reported to be ~0.08%^17^, widespread application of CT angiography improved the ability to diagnose ICAD and ISMAD at initial admission.

However, because of relatively little data for management and evaluation surrounding IVAD, existing studies failed to provide comprehensive analysis for the management, evaluation, prevalence, as well as natural course of the disease.

Therefore, we collected and analyzed current evidence on spontaneous IVAD with the aim of providing quantitative pooled data for the natural course and treatment standardization of the disease. Our primary outcomes were to determine the difference of prevalence, risk factors, and characteristics in different spontaneous IVAD.

## Methods

### Search strategy and study selection

A systematic search of the PubMed, Embase, the Cochrane Library, and Web of Science databases was conducted up to 1 June 2022, for relevant studies, using a search strategy developed by a medical information specialist that involved controlled vocabulary terms and related keywords: (‘Celiac Arteryʼ [Mesh] OR ‘visceral arteryʼ [Title/Abstract]) AND (‘Dissectingʼ [Mesh] OR ‘dissectionʼ[Title/Abstract]). The search was limited to English language articles. Two assessors independently screened the titles and abstracts of the studies. Full texts of relevant studies were obtained for further evaluation. Full texts of related references were also obtained for review. Reference lists of retrieved articles and relevant reviews were manually searched for additional studies. This study reported the included observational clinical studies in accordance with the Meta-analysis of Observational Studies in Epidemiology standard^18^.

### Selection criteria

We included studies if they met the following criteria: (1) patients diagnosed with IVAD; (2) clinical studies that investigated the natural course, treatment, classification, and outcomes of IVAD; (3) studies that reported sufficient data for our pooled analysis.

Studies will be excluded if they meet the following criteria: (1) patients suffered aortic dissection; (2) experimental trials on animals or nonhuman studies; (3) abstracts, letters, editorials, expert opinions, reviews, case reports were excluded; (4) studies with sample size of less than 10 patients; (5) studies without sufficient data or did not meet our including criteria were excluded.

### Quality assessment and data extraction

Two reviewers assessed the quality of each included study. Cohort studies were assessed using the 9-star Newcastle-Ottawa scale (NOS)^19^. The total NOS scores of each cohort study were displayed in the characteristics table (Table 1). The scores were judged according to the three aspects of the NOS of evaluation: selection, comparability, and outcome between the case group and control group. A study with a NOS score greater than or equal to 6 is considered experiencing good quality. In addition, the risk of bias for each studies and the overall risk of bias across all studies were evaluated and shown with figures generated by RevMan 5.2 software^25^. For case series, we used an 18-item tool with the modified Delphi technique^26^ (Table S1, Supplemental Digital Content 1, http://links.lww.com/JS9/A93) and the results of the quality assessment for case series were shown in Table S2, Supplemental Digital Content 1, http://links.lww.com/JS9/A93. Disagreement during assessment was discussed and resolved by a third reviewer.

**Table 1:** The Characteristics of Included Cohort Studies for this Meta-Analysis.

								Treatment			
/References	Country	Study period	Sample size	Age (mean±SD, range)	Indications for intervention	Follow-up time	Coexisting conditions or risk factors	Con. (%)	OS (%)	Endo. (%)	NOS score
Jung *et al*.^6^	Korea	1999−2008	37	58.5 (41−72)	Bowel ischemia	461 (0–2791) days	Renal artery dissection (13.0%)	25	25	50	7
Kang *et al*.^7^	Korea	2006−2018	16	51.7±7.9	Persistent symptoms	33.3 (1.0–118.9) months	Smoking (18.7%) Hypertension (25%) CVD (12.5%) DM (6.3%)	50	0	50	7
Kim *et al*.^10^	Korea	2012−2017	21	56.5±12.7	Persistent symptoms	42.2±47.4 weeks	Smoking (42.9%) Hypertension (38.1%) Hyperlipidemia (19.0%) DM (9.5%) Cancer (14.3%)	100	0	0	7
Kim *et al*.^11^	Korea	2008−2017	28	51.8±10.0	Persistent symptoms	22.0+20.0 months	Hypertension (42.9%) DM 1 (3.6%) Dyslipidemia (7.1%) Smoking (42.9%) Cancer (3.6%) Median arcuate ligament syndrome (3.6%) BMI ≥30 kg/m^2^ (3.6%)	100	0	0	6
Loeffler *et al*.^20^	US	2003−2015	227	55±12.5	Bowel ischemia, AD, DVT	18 (7−45) months	Hypertension (45.1%) CAD (9.3%) Connective tissue disorder (1.9%) Smoking history (50.6%)	43	3	52	7
Peng *et al*.^21^	China	2012−2019	26	53.2±8.8 (36−68)	Bowel ischemia	28.2±14.7 months	Hypertension (50%) DM (11.5%) Dyslipidemia (11.5%) Any smoking (38.5)	46	0	54	7
Tanaka *et al*.^15^	Japan	200−2016	39	52 (49−61)	Bowel ischemia, Rupture	11 (2–45) months	Hypertension (28.6%) Dyslipidemia (21.4%) Any smoking (92.9%) Current smoking (35.7%)	82	7.7	10.3	7
Verde *et al*.^22^	USA	2004−2010	38	60 (30−84)	Pain, nausea, and vomiting, AD	24.77 (0−110.7) months	Smoking (24%) Hypertension (53%)	87.2	5.1	7.7	7
Zettervall *et al*.^23^	US	2006−2014	25	57±13	Bowel ischemia, AD	2.5 (1.2−4.3) years	Any smoking (56%) Current smoker (16%) Hypertension (48%) CAD (8%) DM (12%)	52	12	8	
Zhou *et al*.^24^	China	2013−2020	15	49.5±7.0 (40−61)	Bowel ischemia	12 (1−20) months	Hypertension (26.7%) Dyslipidemia (46.7%)	100	0	0	6

AD, artery dissection; CA, celiac artery; CAD, coronary artery disease; Co, conservative; CVD, cerebrovascular disease; DM, diabetes mellitus; DVT, deep vein thrombosis; Endo, endovascular; NOS, Newcastle-Ottawa Scale; NR, no report. 472; OS, open surgery; pts, patients.

Baseline characteristics were extracted from the included studies using a standardized extraction form. Key characteristics included country, study period, sample size, mean age, coexisting conditions or risk factors, follow-up, and treatment. Two authors independently collected data from the included studies. Any disagreement was handled by discussion with a third reviewer.

### Data synthesis and statistical methods

The data of comparable outcomes between spontaneous IVAD, ICAD, and ISMAD were combined-analyzed using standard statistical procedures in RevMan 5.2^25^ and the proportion or incidence of events was pooled analyzed with Stata 12.0^27^. For dichotomous data, odds ratios (ORs) were calculated, while for continuous data mean differences (MDs) were obtained. Values of proportion outcomes were expressed as proportions and 95% CIs and then transformed into quantities according to the Freeman–Tukey double arcsine transformation^27^.

Heterogeneity between studies was evaluated using the *χ* ^2^-based *Q* statistical test^28^; *P*_*h*_ values, *I* ^2^ statistics, and range values (0–100%) were calculated to quantify heterogeneity. *P*_*h*_≤0.10 indicated significant heterogeneity^29^, and pooled estimates were obtained using a random-effect model (the DerSimonian and Laird method^30^). If statistical heterogeneity was not found (*P*_*h*_>0.10), a fixed-effect model (the Mantel–Haenszel method^31^) was used. The effect sizes of the outcome measures were considered statistically significant if the 95% CIs of pooled ORs did not overlap with 1, or if the 95% CIs of pooled MDs did not overlap with 0.

Finally, Begg’s funnel plot was used to detect publication bias. If the shape of funnel plots revealed no obvious evidence of asymmetry, we considered that there was no obvious publication bias. All statistical analyses were performed using standard statistical procedures provided in Stata 12.0^27^.

This work has been reported in line with Preferred Reporting Items for Systematic Reviews and Meta-Analyses (PRISMA)^32^ (Table S3, Supplemental Digital Content 2, http://links.lww.com/JS9/A94) and Assessing the methodological quality of systematic reviews (AMSTAR) Guidelines^33^ (Table S4, Supplemental Digital Content 3, http://links.lww.com/JS9/A95).

## Results

### Included studies, characteristics, and quality assessment

**Figure 1:**
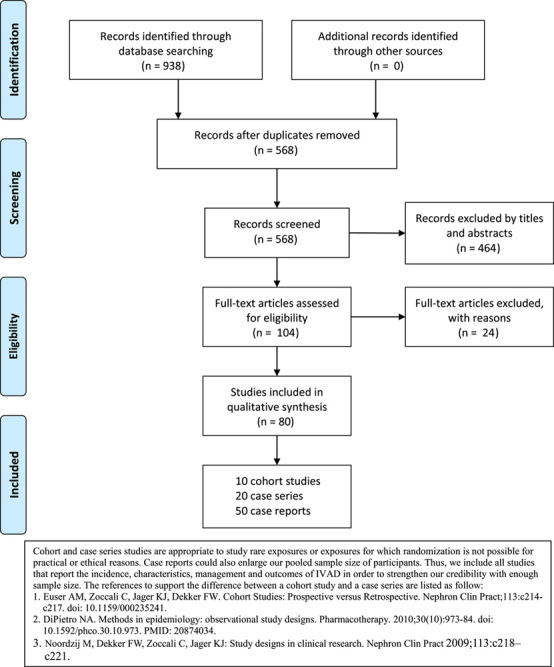
PRISMA flow diagram of literature search and selection of included studies for this meta-analysis.

A total of 938 records of citations were initially identified through database searching; after removing duplicates, 568 of records were reserved for further review. Out of which, 464 records were excluded via screening the titles and abstracts and then 104 articles were assessed for eligibility. After reading the full texts, 24 studies were excluded further as displayed in Figure 1. Eventually, 80 reports (10 cohort studies^6^,^7^,^10^,^11^,^15^,^20^–^24^, 20 case series^8^,^9^,^12^–^14^,^16^,^34^–^47^, and 50 case reports^4^,^5^,^36^,^48^–^94^) with 1040 patients were included in the qualitative synthesis.

**Figure 2:**
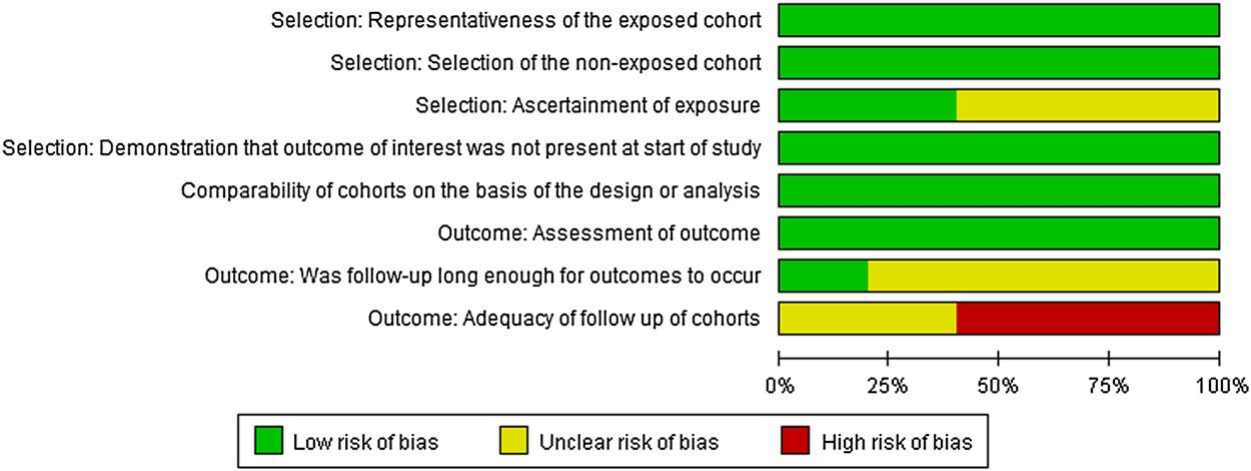
Risk of bias graph: review authors’ judgments about each risk-of-bias item presented as percentages across all included studies.

**Figure 3:**
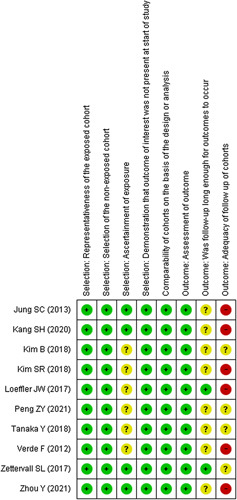
Risk of bias summary: review authors’ judgments about each risk-of-bias item for each included study.

According to our definitions, there was no poor quality in the cohort studies included in this analysis. Additionally, risk-of-bias graphs were generated to further identify the risk of bias of the including cohort studies. The risk of bias for each study was presented as a percentage across all included studies, and the risk-of-bias item for each included study was displayed (Figs. 2 and 3). The risk-of-bias graphs indicated a generally low risk of selection and comparability. In addition, all studies experienced a low risk of bias in the ‘assessment of outcomesʼ item. A high risk of bias was mainly observed in the ‘adequacy of follow-up of cohortsʼ. Unclear risk of bias was mainly observed in ‘adequacy of follow-up of cohortsʼ and ‘follow-up long enough for outcomes to occurʼ.

### Prevalence of dissection in abdominal spontaneous IVAD

**Figure 4:**
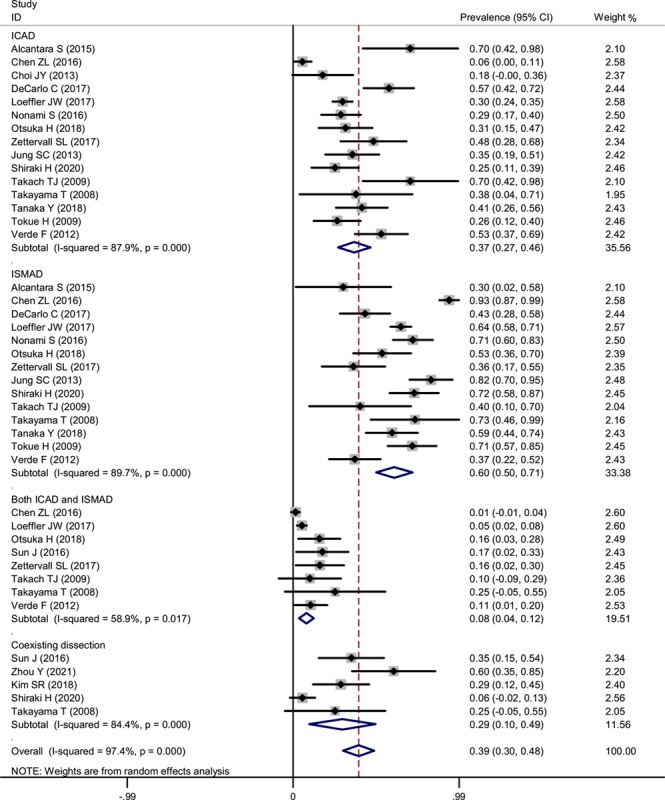
Forest plot showing the pooled prevalence of dissection in abdominal spontaneous IVAD. ICAD, isolated celiac artery dissection; IVAD, isolated visceral artery dissection; ISMAD, isolated superior mesenteric artery dissection.

We analyzed the prevalence of dissection location in IVAD by pooling our data. The pooled results indicated that in IVAD, ISMAD was more prevalent, followed by ICAD. The pooled estimate for the prevalence of ICAD was 37% (95% CI: 27–46%), and the prevalence of ISMAD (60%; 95% CI: 50–71%) was nearly two-fold of ICAD. In addition, only 8% (95% CI: 0.04–0.12) patients experienced a dissection in both CA and SMA. A total of 29% patients also experienced coexisting dissection such as proper hepatic artery, splenic artery, and inferior mesenteric artery (Fig. 4).

### Prevalence of the present of symptoms in IVAD

**Figure 5:**
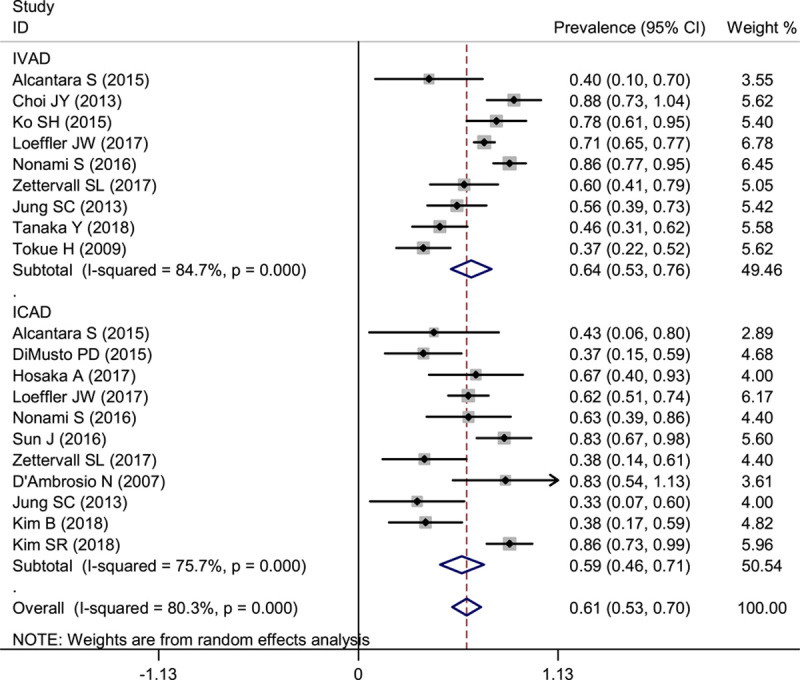
Forest plot showing the prevalence of the present of symptoms in ICAD and IVAD patients. ICAD, isolated celiac artery dissection; IVAD, isolated visceral artery dissection.

A total of 11 and 9 studies evaluated the prevalence of symptoms in ICAD and IVAD, respectively. As our collected data shows 137 of 229 ICAD patients and 313 of 469 IVAD patients experienced symptoms. The prevalence of ICAD and IVAD was 59% (95% CI: 46–71%) and 64% (95% CI: 53–76%), respectively (Fig. 5).

### Prevalence of gastrointestinal (GI) ischemia in abdominal spontaneous IVAD

**Figure 6:**
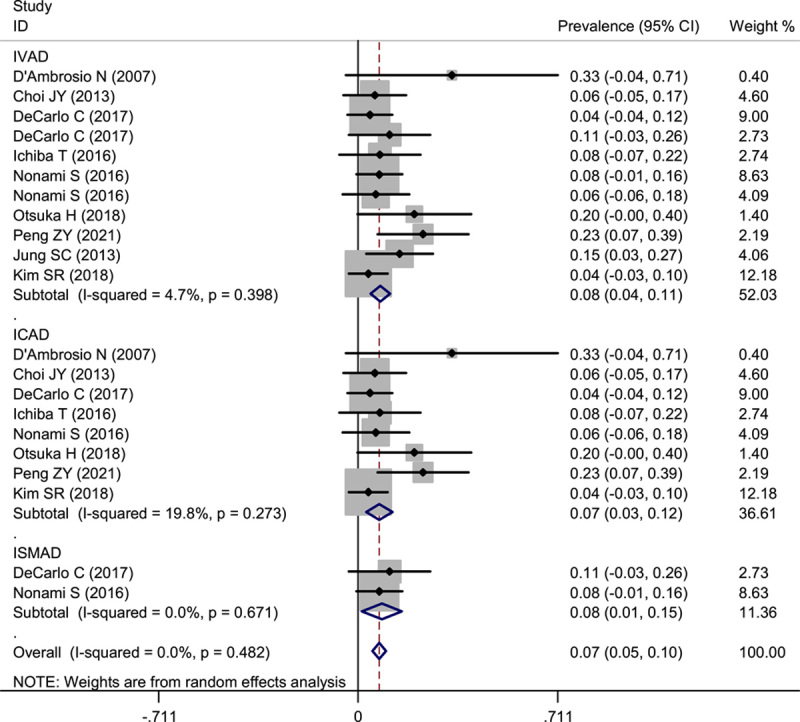
Forest plot of showing the pooled prevalence of GI ischemia in abdominal spontaneous IVAD. ICAD, isolated celiac artery dissection; IVAD, isolated visceral artery dissection; ISMAD, isolated superior mesenteric artery dissection; GI, gastrointestinal.

We pooled analyzed the prevalence of GI ischemia in IVAD. As shown in Figure 6, the pooled estimate for the prevalence of GI ischemia was similar in IVAD (8%; 95% CI: 4–11%), ICAD (7%; 95% CI: 3–12%), and ISMAD (8%; 95% CI: 1–12%).

### Prevalence of atherosclerosis (AS) risk factors and treatment in IVAD

We explored AS risk factors of spontaneous IVAD. Our pooled analysis found hypertension and were smoking the top two conditions in both spontaneous IVAD and ICAD patients, with proportion of 43% (95% CI: 36–51%), 41% (95% CI: 28–54%), and 32% (95% CI: 27–38%), 44% (95% CI: 30–57%), respectively (Table 2). In addition, any other risk factors of IVAD were diabetes mellitus (7%), alcohol consumption (22%), and cerebrovascular disease (12%), respectively. Any other risk factor of ICAD we explored included diabetes mellitus (8%), cerebrovascular disease (13%), vasculitis (30%), and dyslipidemia (13%). For treatment, the majority of ICAD patients received observation (73%; 95% CI: 46–99%). Forty-nine percent and 28% of ICAD patients received conservative and endovascular treatment, respectively. However, 41 and 46% of IVAD patients received observation and conservative treatment. Only 8 and 7% IVAD patients received open surgery and endovascular treatment. In addition, the reintervention rate was much lower in ICAD (8%; 95% CI: 1–21%) than IVAD (24%; 95% CI: 13–36%). The pooled estimate for the prevalence of male patients in ICAD and IVAD was 73% (95% CI: 52–93%) and 80% (95% CI: 72–89%), respectively (Table 2).

**Table 2:** The Prevalence of AS Risk Factors and Treatment in ICAD and IVAD.

	ICAD				IVAD			
			Pooled results				Pooled results	
Outcomes	Number of studies	Patients (*n*/*N*)	Prevalence	95% CI	Number of studies	Patients (*n*/*N*)	Prevalence	95% CI
AS risk factors								
Hypertension	16	94/278	0.32	0.27–0.38	11	234/580	0.43	0.36–0.51
Diabetes mellitus	7	13/153	0.08	0.03–0.13	7	24/273	0.07	0.04–0.10
Smoking	14	106/259	0.44	0.30–0.57	8	167/468	0.41	0.28–0.54
Alcohol consumption					1	16/72	0.22	0.13–0.32
CVD	2	3/24	0.13	−0.01–0.26	1	2/17	0.12	0.04–0.27
Vasculitis	3	3/47	0.30	0.02–0.58				
Dyslipidemia	8	22/157	0.13	0.05–0.20				
Treatment								
Observation	2	13/19	0.73	0.46–0.99	5	69/315	0.41	0.07–0.76
Conservative	7	65/127	0.49	0.22–0.75	8	248/508	0.46	0.24–0.68
Open surgery					7	43/498	0.07	0.04–0.11
Endovascular	7	43/134	0.28	0.10–0.46	8	29/311	0.08	0.04–0.13
Reintervention	8	12/121	0.08	0.01–0.21	3	25/99	0.24	0.13–0.36
Male	16	221/399	0.73	0.52−0.93	11	429/582	0.80	0.72–0.89

AS, atherosclerosis; CVD, cerebrovascular disease; ICAD, isolated celiac artery dissection; IVAD, isolated visceral artery dissection.

### Comparison of spontaneous ICAD versus ISMAD

1. AS risk factors
  We also compared the prevalence of AS risk factors between ICAD and ISMAD patients. Our pooled results found no significant difference in smoking (OR: 1.67; 95% CI: 0.69−4.01; *P=*0.25), hypertension (OR: 0.74; 95% CI: 0.36−1.52; *P=*0.41), dyslipidemia (OR: 0.87; 95% CI: 0.32−2.36; *P=*0.79), and diabetes (OR: 2.09; 95% CI: 0.66−6.62; *P=*0.21), respectively (Table 3).
2. Dissection characteristics and outcomes

**Table 3:** The AS Risk Factors of Spontaneous ICAD Versus ISMAD.

		Pooled results		
Risk factors	Number of patients	OR	95% CI	*P*
Smoking	99	1.67	0.69−4.01	0.25
Hypertension	141	0.74	0.36−1.52	0.41
Dyslipidemia	108	0.87	0.32−2.36	0.79
Diabetes	131	2.09	0.66−6.62	0.21

AS, atherosclerosis; ICAD, isolated celiac artery dissection; ISMAD, isolated superior mesenteric artery dissection; OR, odds ratio.

Compared to ISMAD, ICAD had a shorter dissection length with a pooled MD of −34.44 mm (95% CI: −48.62 to −20.26; *P*<0.0001). In addition, no significant difference was also found in clinical outcomes including failure of medical therapy, recurrent dissection, recurrence of symptoms, death, and remodeling. We compared the computed tomography (CT) findings between ICAD and ISMAD after follow-up, and found ICAD more apt to progress after their first therapy (OR: 2.84; 95% CI: 1.02−7.87). No significant difference was observed in other CT findings (Table 4).

**Table 4:** The Dissection Characteristics and Outcomes of Spontaneous ICAD Versus ISMAD.

		Pooled results		
Names of outcomes	Number of patients	Effect estimate	95% CI	*P*
Dissection characteristics				
Dissection Length (mm)	98	MD −34.44	−48.62 to −20.26	<0.0001
Diameter (mm)	42	MD 0.00	−0.17 to 0.17	1.0
Outcomes				
Failure of Medical Therapy	38	OR 0.23	0.01−6.01	0.38
Recurrent Dissection	30	OR 0.63	0.08−5.17	0.66
Recurrence of Symptoms	30	OR 1.00	0.14−7.10	1.0
Death	30	OR 2.14	0.08−57.06	0.65
Remodeling	30	OR 1.00	0.23−4.31	1.0
CT Finding				
No Change	140	OR 0.74	0.36−1.53	0.41
Partial Improvement	140	OR 1.25	0.55−2.87	0.59
Regression	140	OR 0.52	0.17−1.57	0.25
Progression	140	OR 2.84	1.02−7.87	0.05
Short-term change				
Dissection length (mm)	13	MD −18.60	−27.18, −10.02	<0.0001
Dissection diameter (mm)	13	MD 5.30	−3.46, 14.06	0.24
Stenosis degree (%)	13	MD 6.00	−30.53, 42.53	0.75
Long-term change				
Dissection length (mm)	13	MD −14.8	−55.76, 26.16	0.48
Dissection diameter (mm)	13	MD 1.00	−15.29, 17.29	0.90
Stenosis degree (%)	13	MD −24.6	−64.53, 15.33	0.23

AS, atherosclerosis; ICAD, isolated celiac artery dissection; ISMAD, isolated superior mesenteric artery dissection; MD, mean difference; OR, odds ratio.

We also compared short-term and long-term changes between ICAD and ISMAD. As Table 4 shows, compared to ISMAD, ICAD had less short-term change in dissection length with a pooled MD of −18.60 mm (95% CI: −27.18 to −10.02; *P*<0.0001). No significant difference was found in the short-term change of dissection diameter (MD 5.30 mm; 95% CI: −6.71 to 6.35; *P=*0.24) and stenosis degree (MD 6.0%; 95% CI: −30.53 to 42.53; *P=*0.75). Similarly, there was no significant difference in long-term change of dissection length (MD: −14.8 mm; 95% CI: −55.76 to 26.16; *P=*0.48), dissection diameter (MD: 1.00 mm; 95% CI: −15.29 to 17.29; *P=*0.90), and stenosis degree (MD: −24.6%; 95% CI: −64.53 to 15.33; *P=*0.23), respectively.

### Publication bias

**Figure 7:**
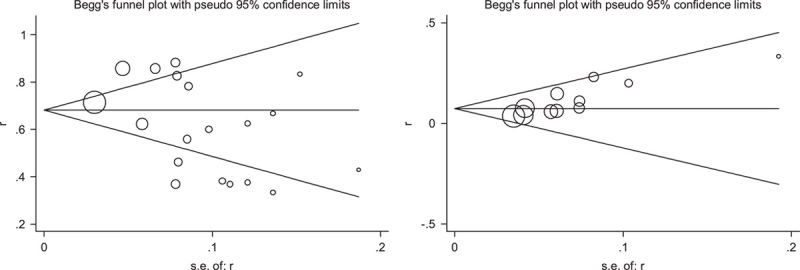
Beeg’s funnel plots showing the publication bias of the prevalence of symptom present (*P=*0.051; left) and gastrointestinal ischemia (*P=*0.679; right) in isolated visceral artery dissection.

Begg’s funnel plots were conducted for assessing the publication bias of included literatures and we could roughly assess the publication bias by seeing whether their shapes were of any obvious asymmetry. According to Figure 7, no clear evidence of publication bias was observed in the prevalence of symptom present (*P=*0.051) and GI ischemia (*P=*0.679) in IVAD.

## Discussion and conclusions

Spontaneous IVAD, which mainly affected the celiac artery and superior mesenteric artery , was defined as dissection of a visceral artery without acute aortic dissection^35^. Spontaneous IVAD is relatively rare and causes various symptoms, including acute abdominal pain, back pain, nausea, and vomiting. It can be catastrophic when it is complicated with intestinal necrosis or rupture. Given the rare previous reports included small numbers of patients, and data on its etiology, clinical features, management, hospital course, and outcomes are scarce. Spontaneous IVAD was reportedly observed in middle-aged men with comorbidities, such as hypertension and smoking^23^,^95^, suggesting that SIVAD might be associated with AS.

The study by Yamaguchi *et al*.^96^ recently indicated that symptomatic spontaneous IVAD patients should be hospitalized because some of those might experience organ ischemia or aneurysm formation. Endovascular intervention was a feasible treatment for complications of IVAD^96^. At present, the etiology behind spontaneous IVAD is unknown and is not well studied.

Therefore, we identified 80 reports with 1040 patients in order to evaluate the prevalence of spontaneous IVAD as well as ICAD and ISMAD in abdominal visceral artery dissection and further found any possible risk factors of the etiology behind spontaneous IVAD. In addition, we compared the clinical features of ICAD to ISMAD and found the clinical distinction of ICAD to other abdominal visceral artery dissection. Our pooled results indicated 72% of male in spontaneous ICAD, which was lower than the report in Cavalcante *et al*.^97^. Regarding the risk factors, our pooled analysis found smoking, hypertension, and vasculitis were the top three conditions in spontaneous ICAD patients, with proportions of 44, 32, and 30%, respectively. The majority of ICAD received observation (58%) and conservative treatment (52%) in our included studies. A total 60% of spontaneous ICAD cases were diagnosed with symptoms. It was observed that ICAD leads to GI ischemia more than ISMAD and IVAD (16 versus 8 and 11%). In addition, when compared to ISMAD, ICAD had several differences in clinical features, such as shorter dissection length, more proportion of Sakamoto II, higher progression, and less change in dissection length in CT finds after treatment. In addition, we also found many differences between symptomatic and asymptomatic ICAD. When compared to asymptomatic ICAD, symptomatic ICAD patients was significantly younger, existed more diabetes, experienced longer dissection length, received less conservative treatment, and more endovascular intervention.

Though our pooled results showed no significant difference in death between asymptomatic and symptomatic ICAD, patient selection and timing of intervention were crucial in the management of ICAD. The ESVS Guidelines suggested that endovascular or open surgery might be considered for patients who had bowel ischemia or were not responding to medical therapy^98^. Galastri *et al*.^40^ suggested that immediate surgical or endovascular treatment is required in the presence of bowel necrosis or aneurysm rupture. In the elective setting, refractory abdominal pain, celiac trunk aneurysm greater than 2 cm, compression of the true lumen, and suspected bowel ischemia were suggested as possible criteria for endovascular treatment. This is similar to the study from Sun *et al*.^46^, where all patients with aneurysmal dilatation underwent intervention.

There existed several limitations in this meta-analysis. First, in our included studies, the number of comparative cohort studies was small, and many were case series. Though we included only case series with sample size larger than 10 and performed sensitivity analysis to strengthen the robustness of our results, the nature deficiencies of retrospective studies always might impair the strength of evidence of our results. Second, the follow-up length varied in each study, and the relatively high proportion of patients lost in the follow-up affected the power of the long-term results. Kang *et al*.^7^ reported the mid-term to long-term outcomes of conservative management and endovascular intervention of ICAD treatment with a median follow-up of 33.3 (range: 1.0−118.9) months and suggested that early intervention in symptomatic SICAD patients may be necessary in over 50% of patients, and endovascular stenting has durable long-term outcomes. However, this study had small sample size with 16 patients and 8 patients in each cohort, which may lead to any risk bias. Third, most series were from China and Japan, very few were from the USA, and even fewer were from Europe. There is a significant difference between Japanese and Western populations in terms of sodium intake, which is known to be associated with a higher incidence of hypertension in Japan^15^. In addition, the rate of smokers in Japan is still higher than that in other developed countries^15^. As our results, hypertension and smoking was two mainly risk factor of IVAD. Thus, this limitation may lead to a risk of bias to our results. Finally, the strategy and duration of conservative management among the included studies were also different, which might have a great impact on the clinical outcomes.

In clinical practice, as spontaneous IVAD is predominantly male and smoking as well as hypertension are the top two conditions in the majority of IVAD patients who always seem healthy, the application of CT angiography should be popularized at initial admission for this population, which could improve the ability to diagnose ICAD and ISMAD. Considering the safety and effectivity of observation and conservative treatment for the majority of patients, this treatment strategy should be suggested.

In conclusion, spontaneous IVAD was male predominant and ISMAD was most prevalent followed by ICAD. Smoking and hypertension were the top two conditions in both spontaneous IVAD and ICAD patients. The majority of patients diagnosed with IVAD received observation and conservative treatment and experienced a low proportion of reintervention or progression, especially for ICAD patients. In addition, ICAD and ISMAD had several differences in clinical features and dissection characteristics. Future studies with a large sample size and long follow-up are required to clear the management, long-term outcome, and risk factors of IVAD prognosis.

## Ethical approval

Ethical approval is not applicable.

## Sources of funding

There is no funding for this work.

## Author contribution

The authors on this paper all participated in the study design. All authors read, critiqued, and approved the manuscript revisions as well as the final version of the manuscript. Also, all authors participated in a session to discuss the results and consider strategies for analysis and interpretation of the data before the final data analysis was performed and the manuscript written.

## Conflicts of interest disclosure

The authors declare no relevant conflict of interest.

## Research registration unique identifying number (UIN)

Research registry.

UNI: reviewregistry1463.

Hyper link: https://www.researchregistry.com/browse-theregistry#registryofsystematicreviewsmetaanalyses/?view_13_search=1463&view_13_page=1

## Guarantor

Yan Shi and Jiangzhou Guo.

## Provenance and peer review

Not commissioned, externally peer peer-reviewed.

## Supplementary Material
