# Supplementary material for: Comparative analysis of prevalence, evaluation, management, and rehabilitation outcome of spontaneous isolated visceral artery dissection: a systematic review and meta-analysis of 80 reports
Source: Int J Surg. 2023 Mar 24;109(3):469–80. doi: 10.1097/JS9.0000000000000301 (PMC10389521; doi:10.1097/JS9.0000000000000301)
Supplement: Supplementary file 1 [file js9-109-469-s001.docx]

**Table SI.** Major components of the tools for assessing case series.

| **Development organization** | **Major components** | **Judgment** |
| --- | --- | --- |
| Carmen Moga and colleagues | 1. Is the hypothesis/aim/objective of the study clearly stated? | 1. Yes, Unclear, No |
|  | 2. Are the characteristics of the participants included in the study described? | 2. Yes, Partially reported, No |
|  | 3. Were the cases collected in more than one center? | 3. Yes, Unclear, No |
|  | 4. Are the eligibility criteria (ie, inclusion and exclusion criteria) for entry into the study clearly stated? | 4. Yes, Partially reported, No |
|  | 5. Were participants recruited consecutively? | 5. Yes, Unclear, No |
|  | 6. Did participants enter the study at a similar point in the disease? | 6. Yes, Unclear, No |
|  | 7. Was the intervention of interest clearly described? | 7. Yes, Partially reported, No |
|  | 8. Were additional interventions (cointerventions) reported in the study? | 8. Yes, Unclear, No |
|  | 9. Are the outcome measures established a priori? | 9. Yes, Partially reported, No |
|  | 10. Were the relevant outcomes measured with appropriate objective or subjective methods? | 10. Yes, Unclear, No |
|  | 11. Were the relevant outcomes measured before and after the intervention? | 11. Yes, Unclear, No |
|  | 12. Were the statistical tests used to assess the relevant outcomes appropriate? | 12. Yes, Unclear, No |
|  | 13. Was the length of follow-up reported? | 13. Yes, Unclear, No |
|  | 14. Was the loss to follow-up reported? | 14. Yes, Unclear, No |
|  | 15. Does the study provide estimates of the random variability in the data analysis of relevant outcomes? | 15. Yes, Unclear or partially reported, No |
|  | 16. Are the adverse events related with the intervention reported? | 16. Yes, Partially reported, No |
|  | 17. Are the conclusions of the study supported by results? | 17. Yes, Partially reported, No |
|  | 18. Are both competing interests and sources of support for the study reported? | 18. Yes, Partially reported, No |

**Table S2.** Results of quality assessment of included case series.

| **Study** | **1** | **2** | **3** | **4** | **5** | **6** | **7** | **8** | **9** | **10** | **11** | **12** | **13** | **14** | **15** | **16** | **17** | **18** |
| --- | --- | --- | --- | --- | --- | --- | --- | --- | --- | --- | --- | --- | --- | --- | --- | --- | --- | --- |
| Alcantara S et al. (2015) | Y | Y | N | Y | U | N | Y | N | P | Y | Y | Y | Y | Y | Y | Y | Y | Y |
| Chen ZL et al. (2016) | Y | Y | N | Y | U | N | Y | N | P | Y | Y | Y | Y | Y | Y | Y | Y | Y |
| Choi JY et al. (2013) | Y | Y | N | Y | U | N | Y | Y | Y | Y | Y | Y | Y | Y | Y | Y | Y | Y |
| D'Ambrosio N et al. (2007) | Y | Y | N | Y | U | N | Y | N | Y | Y | Y | Y | Y | Y | Y | Y | Y | Y |
| DeCarlo C et al. (2017) | Y | Y | N | Y | U | N | Y | Y | Y | Y | Y | Y | Y | Y | Y | Y | Y | Y |
| DiMusto PD et al. (2015) | Y | Y | Y | Y | U | N | Y | N | Y | Y | Y | Y | Y | N | Y | Y | Y | Y |
| Galastri FL et al. (2015) | Y | Y | N | Y | U | N | Y | N | P | Y | Y | Y | Y | Y | Y | Y | Y | Y |
| Hosaka A et al. (2017) | Y | Y | N | Y | Y | N | Y | Y | P | Y | Y | Y | Y | Y | Y | Y | Y | Y |
| Ichiba T et al. (2016) | Y | Y | N | Y | U | N | Y | N | Y | Y | Y | Y | Y | Y | Y | Y | Y | Y |
| Ko SH et al. (2015) | Y | Y | Y | Y | U | N | Y | Y | P | Y | Y | Y | Y | U | Y | Y | Y | Y |
| Li S et al. (2017) | Y | Y | N | Y | U | N | Y | N | P | Y | Y | Y | Y | Y | Y | Y | Y | Y |
| Nonami S et al. (2016) | Y | Y | N | Y | U | NY | Y | Y | Y | Y | Y | Y | Y | Y | Y | Y | Y | Y |
| Oh S et al. (2011) | Y | Y | N | Y | U | N | Y | Y | Y | Y | Y | Y | Y | Y | Y | Y | Y | Y |
| Otsuka H et al. (2018) | Y | Y | N | Y | Y | N | Y | N | P | Y | Y | Y | Y | Y | Y | Y | Y | Y |
| Park WJ et al. (2018) | Y | Y | N | Y | U | N | Y | N | Y | Y | Y | Y | Y | Y | Y | Y | Y | Y |
| Shiraki H et al. (2020) | Y | Y | N | Y | Y | N | Y | Y | P | Y | Y | Y | Y | Y | Y | Y | Y | Y |
| Sun J et al. (2016) | Y | Y | N | Y | U | N | Y | Y | Y | Y | Y | Y | Y | Y | Y | Y | Y | Y |
| Takach TJ et al. (2009) | Y | Y | N | Y | Y | N | Y | Y | P | Y | Y | Y | Y | Y | Y | Y | Y | Y |
| Takayama T et al. (2008) | Y | Y | N | Y | U | N | Y | N | Y | Y | Y | Y | Y | Y | Y | Y | Y | Y |
| Tokue H et al. (2009) | Y | Y | N | Y | U | N | Y | Y | Y | Y | Y | Y | Y | Y | Y | Y | Y | Y |

N, Not addressed; P, partially addressed; U, unknown; Y, yes, fully addressed. Items 1 to 18 indicate 18 components of quality assessment for case series.
